# Evolutionary rate covariation analysis of E-cadherin identifies Raskol as a regulator of cell adhesion and actin dynamics in *Drosophila*

**DOI:** 10.1371/journal.pgen.1007720

**Published:** 2019-02-14

**Authors:** Qanber Raza, Jae Young Choi, Yang Li, Roisin M. O’Dowd, Simon C. Watkins, Maria Chikina, Yang Hong, Nathan L. Clark, Adam V. Kwiatkowski

**Affiliations:** 1 Department of Cell Biology, University of Pittsburgh School of Medicine, Pittsburgh, Pennsylvania, United States of America; 2 Center for Genomics and Systems Biology, Department of Biology, New York University, New York, New York, United States of America; 3 Center for Biologic Imaging, University of Pittsburgh School of Medicine, Pittsburgh, Pennsylvania, United States of America; 4 Department of Computational and Systems Biology, University of Pittsburgh School of Medicine, Pittsburgh, Pennsylvania, United States of America; New York University, UNITED STATES

## Abstract

The adherens junction couples the actin cytoskeletons of neighboring cells to provide the foundation for multicellular organization. The core of the adherens junction is the cadherin-catenin complex that arose early in the evolution of multicellularity to link actin to intercellular adhesions. Over time, evolutionary pressures have shaped the signaling and mechanical functions of the adherens junction to meet specific developmental and physiological demands. Evolutionary rate covariation (ERC) identifies proteins with correlated fluctuations in evolutionary rate that can reflect shared selective pressures and functions. Here we use ERC to identify proteins with evolutionary histories similar to the *Drosophila* E-cadherin (DE-cad) ortholog. Core adherens junction components α-catenin and p120-catenin displayed positive ERC correlations with DE-cad, indicating that they evolved under similar selective pressures during evolution between *Drosophila* species. Further analysis of the DE-cad ERC profile revealed a collection of proteins not previously associated with DE-cad function or cadherin-mediated adhesion. We then analyzed the function of a subset of ERC-identified candidates by RNAi during border cell (BC) migration and identified novel genes that function to regulate DE-cad. Among these, we found that the gene *CG42684*, which encodes a putative GTPase activating protein (GAP), regulates BC migration and adhesion. We named *CG42684 raskol* (“to split” in Russian) and show that it regulates DE-cad levels and actin protrusions in BCs. We propose that Raskol functions with DE-cad to restrict Ras/Rho signaling and help guide BC migration. Our results demonstrate that a coordinated selective pressure has shaped the adherens junction and this can be leveraged to identify novel components of the complexes and signaling pathways that regulate cadherin-mediated adhesion.

## Author summary

The establishment of intercellular adhesions facilitated the genesis of multicellular organisms. The adherens junction, which links the actin cytoskeletons of neighboring cells, arose early in the evolution of multicellularity and selective pressures have shaped its function and molecular composition over time. In this study, we used evolutionary rate covariation (ERC) analysis to examine the evolutionary history of the adherens junction and to identify proteins that coevolved with the core adherens junction protein *Drosophila* E-cadherin (DE-cad). ERC analysis of DE-cad revealed a collection of proteins with similar evolutionary histories. We then tested the role of ERC-identified candidates in border cell migration in the fly egg chamber, a process that requires the coordinated regulation of cell-cell adhesion and cell motility. Among these, we found that a previously uncharacterized gene *CG42684*, which encodes a putative GTPase activating protein (GAP), regulates the collective cell migration of border cells, stabilizes cell-cell adhesions and regulates the actin dynamics. Our results demonstrate that components of the adherens junction share an evolutionary history and that ERC analysis is a powerful method to identify novel components of cell adhesion complexes in *Drosophila*.

## Introduction

The adherens junction (AJ) is a multiprotein complex that is essential for intercellular adhesion in metazoa. The core of the AJ is the cadherin-catenin complex. Classical cadherins are single-pass transmembrane proteins with an extracellular domain that mediates calcium-dependent homotypic interactions. The adhesive properties of classical cadherins are driven by the recruitment of cytosolic catenin proteins to the cadherin tail: p120-catenin binds to the juxta-membrane domain and β-catenin binds to the distal part of the tail. β-Catenin recruits α-catenin to the cadherin-catenin complex. α-Catenin is an actin-binding protein and the primary link between the AJ and the actin cytoskeleton [1–3].

The primary function of the AJ is to link actin to intercellular junctions. It is believed that the AJ arose early in the evolution of multicellular metazoans to coordinate epithelial tissue formation and organization [4–7]. The AJ has since evolved to function in a range of physiological and developmental processes, including cell polarity, collective cell migration and cell division [8, 9]. AJ function in these diverse processes requires an array of ancillary regulatory proteins, including kinases, signaling molecules and adaptor proteins [10–14]. Defining the molecular networks that regulate AJ biology is critical to understanding cadherin-mediated adhesion in normal and disease states.

Evolutionary rate covariation (ERC) analysis is a comparative genomic approach that has been used successfully to identify proteins with shared functions in canonical protein complexes and biological processes in prokaryotes, fungi, *Drosophila* and mammals [15–21]. ERC works from the principle that co-functioning proteins would often experience shared changes in selective pressure as they evolve together in different species. Those changes lead to shifts in amino acid substitution rates that are shared by co-functional proteins and which are apparent in their substitution rates over the branches of the species tree along which they evolved. The result is a correlation of substitution rates between the co-functional proteins that we term ERC. An ERC value is calculated as the correlation coefficient between a pair of proteins of their branch-specific evolutionary rates from the phylogenetic tree separating their orthologous sequences from multiple species [19]. Note that proteins exhibiting ERC across a tree could still have very different average substitution rates; it is only the variation of those rates that matters in the correlation. ERC analysis permits the identification of protein-coding genes that evolved in a correlated manner and hence might function in the same pathway or molecular complex. These genes can then be screened by RNAi-based knockdown or similar genetic approaches to validate their role in a relevant biological process. Indeed, ERC-based inference has led to the discovery of many new genes as participants in pathways of interest, such as in the *Drosophila* female post-mating response, connections between human diseases, and the *Drosophila* neuromuscular junction [16, 18, 21]. Each of these studies searched for new functional connections between protein-coding genes by identifying proteins exhibiting ERC with known pathway components.

Border cell (BC) migration in the developing *Drosophila* egg chamber requires coordinated cell adhesion and migration. During BC migration, a group of 6–8 follicular cells delaminate from the anterior most tip of the epithelium and undergo haptotaxis and migrate collectively towards the developing oocyte [22, 23]. The BC cluster consists of migratory BCs and a centrally positioned pair of polar cells (PCs) that signal to BCs and contribute to cluster adherence [23]. BC migration is highly dependent on *Drosophila* E-Cadherin (DE-cad, encoded by *shotgun* (*shg*)) [24–26]. Upregulation of DE-cad is essential for the initial delamination and subsequent migration of BCs since disruption of DE-cad-mediated adhesion affects the ability of BCs to detach from the follicular epithelium (FE) and collectively migrate [24, 25].

We performed ERC analysis of DE-cad to identify proteins that share a common evolutionary history, and therefore may share an overlapping function, with DE-cad and assessed their role in BC migration. Primary components of AJ, α-catenin and p120-catenin, display high ERC values relative to DE-cad and one another, suggesting that these genes and their protein products are co-functional, which is well described in the literature [1, 27]. We show that proteins showing high ERC values with DE-cad are enriched for membrane-associated proteins and proteins that function in E-cadherin-dependent biological processes. We then conducted an RNAi-mediated genetic screen in BCs with 34 high-ranking ERC candidates and identified both novel and known genes that function to regulate DE-cad at cell contacts. Among those, we characterized a GTPase activating protein (GAP) domain encoding gene, *CG42684*, which we have named “*raskol*” after the Russian term “to split”. We show that Raskol colocalizes with DE-cad, regulates DE-cad levels at the BC-BC interface and modulates actin-rich protrusions during BC migration. Our results demonstrate that components of the AJ share an evolutionary history and that ERC analysis is a powerful method to identify novel components of cell adhesion complexes in *Drosophila*.

## Results

### ERC analysis identifies proteins that coevolved with DE-cad

**Fig 1:**
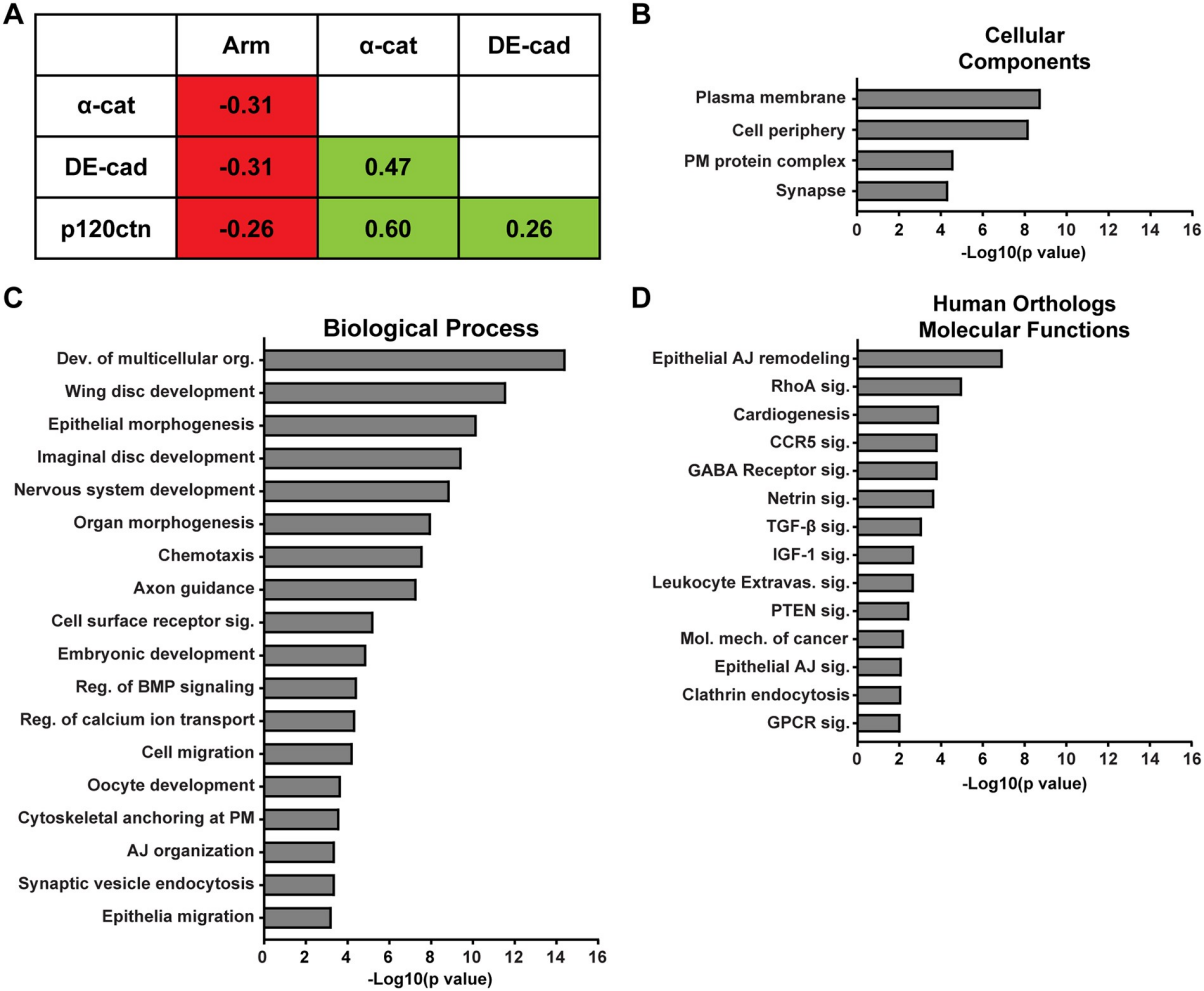
Enrichment analysis of DE-cad ERC candidates. A. ERC analysis revealed an evolutionary relationship between DE-cadherin, α-catenin and p120-catenin. B. DE-cad ERC hits with values ≥ 0.4 were enriched for plasma membrane and cell periphery proteins. C. DE-cad list was enriched for proteins with established roles in regulating biological processes that require cadherin-mediated adhesion. D. Human homologs of the DE-cad ERC regulate epithelial AJ remodeling and multiple canonical pathways including RhoA, TGF-β, PTEN and AJ signaling.

We used ERC analysis to identify novel proteins that regulate DE-cad-mediated adhesion in *Drosophila*. Since ERC signatures are often observed between proteins that function in a molecular complex [15, 16, 18, 19], we evaluated the ERC values of the fly AJ components—DE-cad, Armadillo (*arm*; β-catenin in vertebrates), α-catenin (*α-cat*), p120-catenin (*p120ctn*), Vinculin (*Vinc*) and Canoe (*cno*; afadin in vertebrates). Notably, DE-cad, α-catenin and p120-catenin displayed positive ERC values with each other (Fig 1A). Armadillo, Vinculin and Canoe did not show elevated ERC values relative to DE-cad, α-catenin or p120-catenin. Since Armadillo, Vinculin and Canoe are known to function independently of cadherin-mediated adhesion [28–31], we speculate that alternative selective pressures stemming from their other functional roles have influenced the evolution of these proteins in flies, likely obscuring any ERC signature with primary AJ proteins. Nonetheless, ERC analysis suggested that the AJ components DE-cad, α-cat and p120ctn coevolved to maintain their collective function in cell-cell adhesion. We postulated that other proteins that regulate AJ biology would have similar evolutionary histories to maintain functionality.

We then used ERC analysis to identify proteins with high ERC values relative to the core of the AJ complex, DE-cad (S1 Table). We identified 137 proteins with ERC values of 0.4 or greater, representing the top 1.3% of DE-cad ERC values. Since α-cat had an ERC value of 0.47 relative to DE-cad (Fig 1A), placing their ERC value in the top 0.6% of all protein pairs, we reasoned that proteins with similar or higher ERC values would represent proteins with similar evolutionary histories to DE-cad. Accordingly, the thresholded DE-cad ERC list contained proteins with described roles in AJ regulation such as Hrb98DE [32, 33], PDZ-GEF [34, 35], Baboon [36, 37], CG16952 [38, 39] and Rab5 [40–42] (Table 1). Notably, the majority of ERC identified proteins have not been associated with the AJ and include transcription factors, kinases, GTPase regulatory proteins and calcium channel regulators (Table 1). A previous genomic RNAi screen conducted in *Drosophila* S2 cells [43] and E-Cadherin proximity biotinylation screens in epithelial cells [44, 45] identified multiple hubs of interactors and regulators. Cross referencing the DE-cad ERC list with the hits from these screens revealed only a few common genes such as RhoGAPp190, Rab5, Appl and Stim. This suggests that ERC analysis identified additional DE-cad regulatory components that were undetected in prior genetic or proteomic screens.

**Table 1:** Function of selected conserved proteins identified in DE-cad ERC analysis. *Score from flybase (www.flybase.org) orthologue database. Ratio indicates sequence alignment algorithms that reported significant homology with mammalian homologs.

Protein	ERC value	Function in flies	Homolog(s) (score*)	Homolog function in mammals
Grk	0.62	Growth factor (EGF)	Nrg (1/15)	EGF family receptor ligand
Cac	0.62	Voltage-gated Ca^2+^ channel subunit	Cacna1 (11/15)	Voltage-gated Ca^2+^ channel subunit
Hrb98DE	0.54	mRNA binding	Hnrnpa2b1 (13/15)	RNA binding protein
PDZ-GEF	0.53	Rap1 GEF	Rapgef2 (13/15)	Rap GEF
			Rapgef6 (12/15)	
Rab5	0.52	Rab GTPase; protein trafficking	Rab5c (13/15)	Rab GTPase; protein trafficking
			Rab5b (14/15)	
Stim	0.52	Ca^2+^ channel regulator	Stim1 (13/15)	Ca^2+^ influx regulation
Raskol CG42684	0.50	GTPase activator activity (inferred)	Dab2ip (8/15)	Ras-GAP
			Rasal2 (8/15)	Ras-GAP
			Syngap1 (7/15)	Synaptic Ras-GAP
Babo	0.5	Activin (TGFβ) receptor	Tgfbr1 (14/15)	TGF-β receptor
			Acvr1 (11/15)	
Gug	0.49	Nuclear repressor	Rere (10/15)	Transcriptional repressor
Pdk1	0.46	Kinase; cell signaling	Pdpk1 (11/15)	Kinase; cell signaling
CG16952	0.46	-	Btbd7 (10/15)	Branching morphogenesis
CG11593	0.46	-	Bnip2 (6/15)	Rho GTPase signaling
CG14883	0.44	-	Gde1 (12/15)	Glycerophosphodiester phosphodiesterase

Using gene ontology (GO) based enrichment analysis, we found that the DE-cad ERC hits were enriched for plasma membrane (PM) localized proteins and PM-associated protein complexes (Fig 1B). Additionally, proteins that function in biological processes requiring E-cadherin-mediated adhesion, such as wing disc morphogenesis, imaginal disc morphogenesis, epithelial morphogenesis, cell migration and AJ organization, were significantly overrepresented in the DE-cad ERC list (Fig 1C). Next, we analyzed the molecular functions of the human orthologs of the ERC identified proteins. We found that proteins involved in epithelial AJ remodeling and cancer molecular mechanism were overrepresented (Fig 1D; S2 and S3 Tables). Also, proteins that function in RhoA, CCR5, TGF-β, PTEN and AJ-mediated signalling pathways were enriched (Fig 1D, S2 and S3 Tables). Thus, the DE-cad ERC list contains proteins with established roles in regulating AJs as well as novel candidate proteins.

### RNAi screen in BCs identifies genes that regulate cell-cell adhesion

**Fig 2:**
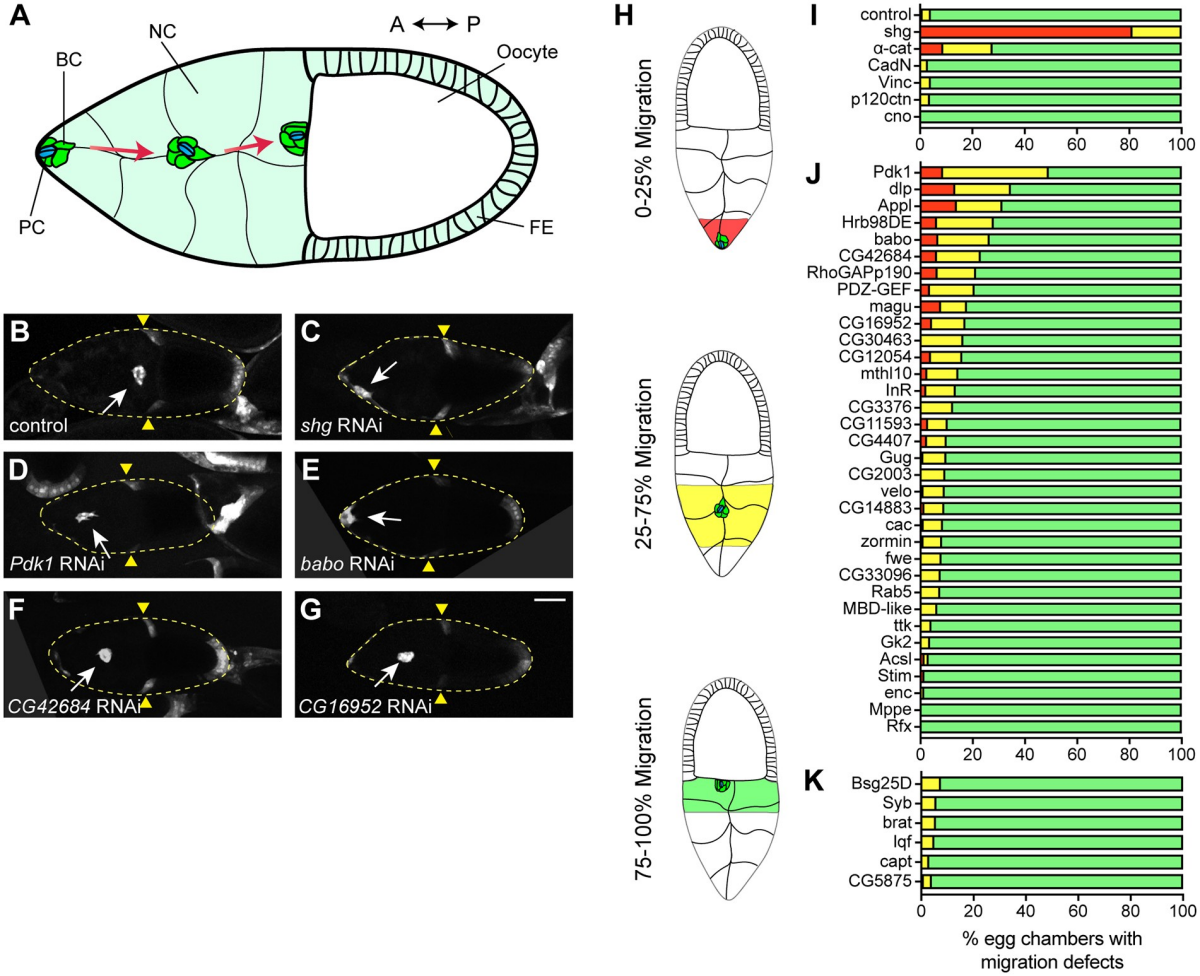
DE-cad ERC genes regulate BC migration. A. Cartoon representation of BC migration during egg chamber development. BC, border cell; PC, polar cell; NC, nurse cell; FE, follicular epithelium; A, anterior; P, posterior. B-G. Representative images of egg chambers expressing UAS-GFP and UAS-RNAi for control (luciferase) (B), *shg* (C), *Pdk1* (D), *babo* (E), *CG42684* (F) or *CG16952* (G) under the control of *slbo*-GAL4. White arrow indicates BC cluster position. Yellow triangles mark FE retraction border. Maximum projections of 20 μm z-stacks are shown. Posterior is to the right in all images. H. Border cell migration scoring classes. I-K. Percentage of egg chambers in each class displaying migration defects in AJ-related genes (I), DE-cad ERC target genes (J) and random negative control genes (K). Red 0–25%, yellow 25–75% and green 75–100%. N values are listed in S5 Table. Scale bar in G is 50 μm and applies to B-G.

To evaluate the function of DE-cad ERC candidates, we conducted an *in vivo* RNAi-based genetic screen in the *Drosophila* egg chamber. We analyzed BC collective cell migration (CCM) because it is regulated by DE-cad and is a powerful system to study the interplay between cell migration and cell-cell adhesion (Fig 2A) [23, 24]. BC detachment from the FE and concomitant CCM requires increased DE-cad expression and loss of DE-cad arrests BC migration [24, 25].

First, we downregulated levels of individual AJ genes by using the GAL4/UAS system to drive UAS-RNAi transgenes in the migrating BC cluster. We used a BC specific driver, *slow border cell* GAL4 (*slbo*-GAL4) [46, 47] to drive expression of a UAS-GFP reporter and a UAS-RNAi transgene targeted against the gene of interest. Stage 10 egg chambers expressing RNAi and GFP in BCs were fixed and scored for BC cluster position along the anterior-posterior migration axis (Fig 2H). In control egg chambers, nearly all BC clusters completed migration and were positioned adjacent to the oocyte (Fig 2B and 2I). In contrast, downregulation of *shg* caused a BC migration failure or delay in all egg chambers (Fig 2C and 2I). BC migratory defects were less severe in egg chambers with reduced expression of *α-cat* compared to *shg*; however, the prevalence of defects was higher than in control egg chambers (Fig 2I). We could not assess the effect of *arm* downregulation since *arm* RNAi expressing flies did not survive to adulthood. The downregulation of *CadN*, *Vinc*, *p120ctn* or *cno* did not cause BC migratory defects (Fig 2I).

Next, we screened 34 genes from the DE-cad ERC list. We focused on genes that are expressed in the ovary [48] and for which an RNAi stock was readily available (S4 Table). The downregulation of target genes displayed a variable range of BC migration defects with 12 genes displaying defects in more than 15% of egg chambers assessed compared to 4% in control (Fig 2J). We also randomly selected and screened six genes whose protein products had either very low ERC values or did not appear in the DE-cad ERC list for migration defects. As expected, we did not observe strong migration defects when these genes were knocked down (Fig 2K). Moreover, the DE-cad ERC genes showed statistically lower average migration than the random negative control genes (Wilcoxon rank sum test, p = 0.0162), supporting the hypothesis that proteins with correlated evolutionary histories share functional characteristics. *Pdk1* knockdown resulted in the most penetrant phenotype with 50% of egg chambers displaying either a failure or delay in BC migration (Fig 2D and 2J). Knockdown of *babo* and *CG42684* caused migration delays similar to *α-Cat* (Fig 2E and 2F). Additionally, knockdown of multiple genes resulted in considerable migration delays relative to the control including *CG16952* (Fig 2G), *Hrb98DE*, *magu*, *InR*, *RhoGAPp190*, *PDZ-GEF* and *CG11593* (Fig 2J). Conversely, knockdown of genes such as *enc*, *mppe* and *rfx* did not affect BC migration (Fig 2J).

**Fig 3:**
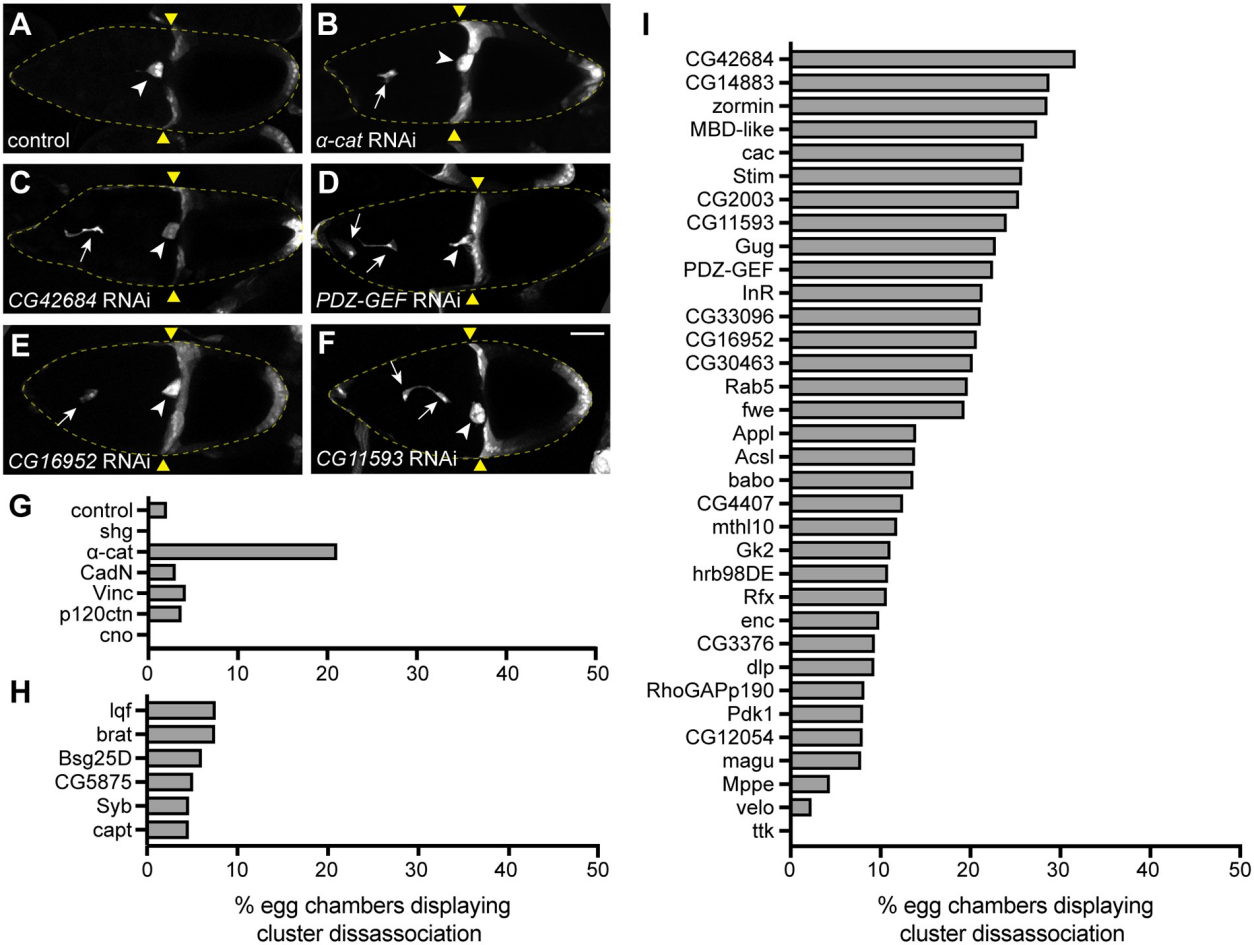
DE-cad ERC genes maintain BC adhesion during migration. A-F. Egg chambers expressing UAS-GFP and UAS-RNAi for control (A), *α-cat* (B), *CG42684* (C), *PDZ-GEF* (D), *CG16952* (E) or *CG11593* (F). White arrows mark disassociated BCs. White arrowheads mark BC cluster adjacent to oocyte. Yellow triangles mark the FE retraction border. Maximum projections of 20 μm z-stacks are shown. G-I. Percentage of egg chambers displaying a cluster disassociation phenotype in AJ genes (G), random negative control genes (H) and DE-cad ERC target genes (I) (I). Scale bar in F is 50 μm and applies to A-F.

While screening *α-cat* knockdown egg chambers, we noticed that about 20% of the egg chambers displayed a cluster disassociation phenotype where one or more BCs had separated from the cluster (Fig 3B and 3G). Since this phenotype is indicative of cell adhesion defects in BCs [24], we scored cluster disassociation for all genotypes. In control BCs expressing luciferase RNAi, disassociated clusters were rarely observed (Fig 3A and 3G). Likewise, downregulation of genes with low ERC values did not show a cluster disassociation phenotype (Fig 3H). Knockdown of *CadN*, *vinc*, *p120ctn* and *cno* also did not cause a penetrant cluster disassociation phenotype (Fig 3G). However, cluster disassociation phenotypes were observed with a number of ERC target genes. *CG42684*, *PDZ-GEF*, *CG16952*, *CG11593*, *zormin*, *cac* and *Rab5* displayed similar or higher cluster disassociation phenotypes compared to *α-cat* (Fig 3C–3F and 3I). Overall, these ERC target genes, chosen for their high DE-cad ERC values, exhibited the cluster disassociation phenotype significantly more often than genes with low DE-cad ERC values (Wilcoxon rank sum test, p = 0.00022). Together, these results demonstrate that highly ranked genes in the DE-cad ERC list contain factors that may regulate cell adhesion during BC collective cell migration.

### Top ERC candidates regulate DE-cad levels in BCs

**Fig 4:**
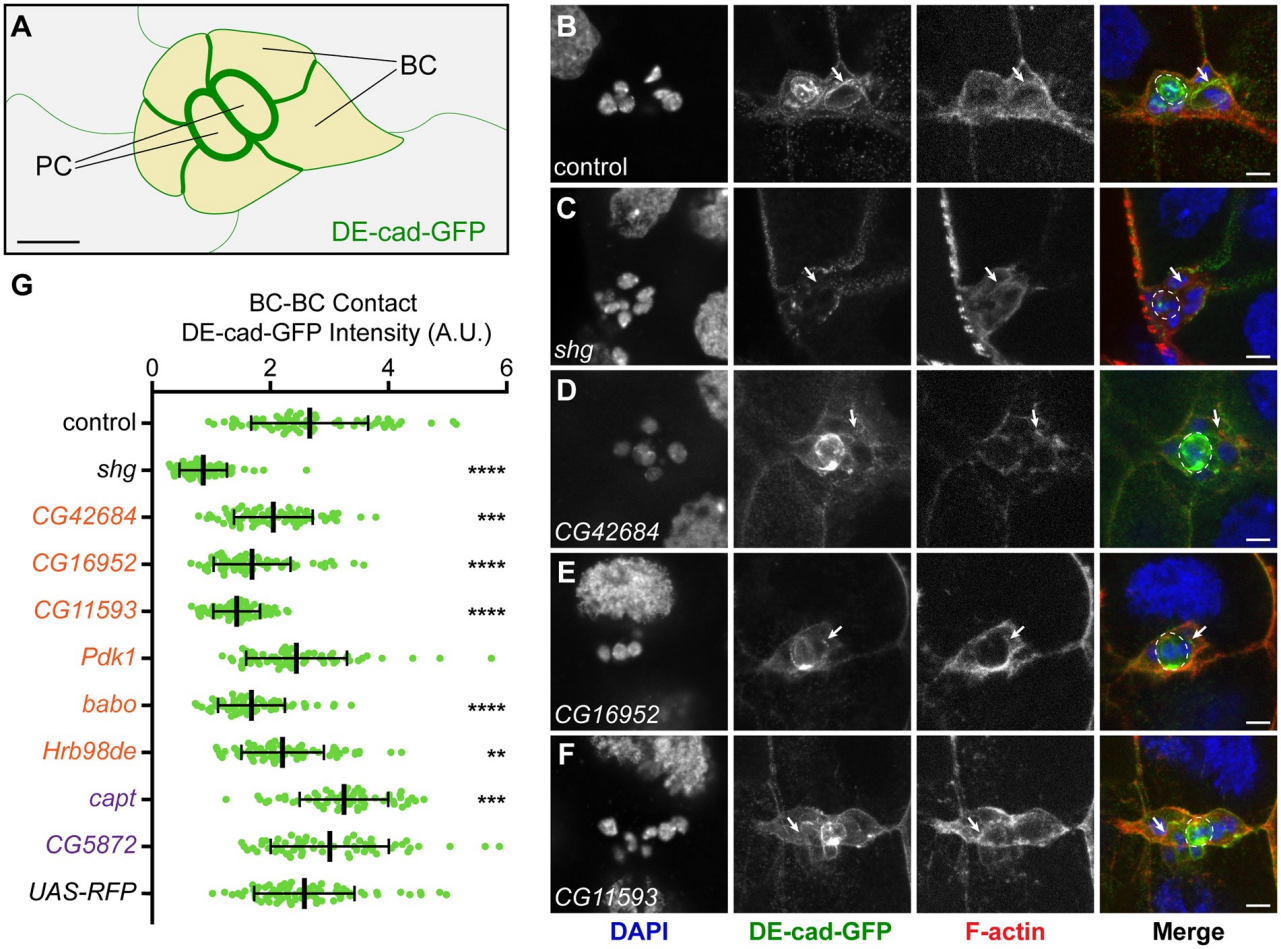
DE-cad ERC genes regulate DE-cad levels at BC cell-cell contacts. A. Cartoon schematic of DE-cad-GFP enrichment at cell-cell contacts in a BC cluster. Enrichment is highest PC-PC and PC-BC, intermediate at BC-BC contacts and lower at BC-NC contacts. B-F. Representative images of BC clusters expressing control (B), *shg* (C), *CG42684* (D), *CG16952* (E) and *CG11593* (F) RNAi under control of *slbo*-GAL4. DAPI (first column; blue in merge), DE-cad-GFP (second column; green in merge), F-actin (third column; red in merge) and merge (fourth column) channels shown. White arrows mark BC-BC contacts. PCs are outlined by a dashed line. Maximum projections of 5 μm z-stacks shown. G. Quantification of DE-cad levels at BC-BC contacts in control (n = 69), *shg* (n = 66), *CG42684* (n = 72), *CG16952* (n = 69), *CG11593*(n = 69), *Pdk1*(n = 66), *babo* (n = 69), *Hrb98DE* (n = 72), *capt* (n = 60), *CG5875* (n = 60) and *UAS-RFP* (n = 75) RNAi-expressing BCs. **** p<0.0001, *** p<0.001, ** p<0.01. Middle bar is the mean and error bars represent SD. Controls are in black, DE-cad ERC hits are in orange and random ERC hits in purple. Scale bar in A and Merge in B-F is 10 μm.

Since knockdown of a subset of DE-cad ERC genes disrupted BC migration, we questioned if they might regulate DE-cad at BC contacts. To test this, we quantified DE-cad levels along cell-cell contacts between BCs in RNAi-expressing clusters (Fig 4A). We used a DE-cad-GFP knock-in stock that express DE-cad at endogenous levels [49] and drove RNAi constructs under the control of *slbo*-GAL4. In control egg chambers where either luciferase RNAi or RFP were expressed, DE-cad-GFP was enriched at BC-BC contacts (Fig 4B, S1F Fig). As expected, *shg* RNAi expression severely reduced DE-cad levels in the BC cluster (Fig 4C). Knockdown of *CG42684* (Fig 4D), *CG16952* (Fig 4E), *CG11593* (Fig 4F), *babo* (S1B Fig) and *Hrb98DE* (S1C Fig) caused a significant reduction in DE-cad levels at BC-BC contacts. Interestingly, knockdown of *Pdk1*, a kinase in the PI3K pathway [50], did not affect DE-cad levels in BCs even though it displayed the most prominent migration defect (S1A Fig). We also analyzed two proteins with low ERC values relative to DE-cad–*CG5872*, an uncharacterized gene, and *capt*, which encodes Capulet, an actin-binding protein that inhibits actin polymerization [51]–that did not disrupt BC migration or adhesion when knocked down (Figs 2K and 3H). Knockdown of *CG5872* did not affect DE-cad levels at BC-BC contacts (S1E Fig, Fig 4G), but knockdown of *capt* increased DE-cad levels at BC-BC contacts (S1D Fig, Fig 4G). Loss of *capt* has been shown to promote F-actin accumulation [51–53] and cadherins are stabilized by actin at cell-cell contacts [54], possibly explaining the increase in DE-cad at BC contacts. Notably, overexpression of DE-cad does not impact BC migration [24], consistent with *capt* RNAi expression not affecting BC migration or cluster adhesion (Figs 2K and 3H). Collectively, these results indicate that a subset of proteins with high ERC values relative to DE-cad, many of which were previously not associated with cadherin function, regulate DE-cad levels at BC-BC contacts.

### Raskol colocalizes with DE-cadherin

**Fig 5:**
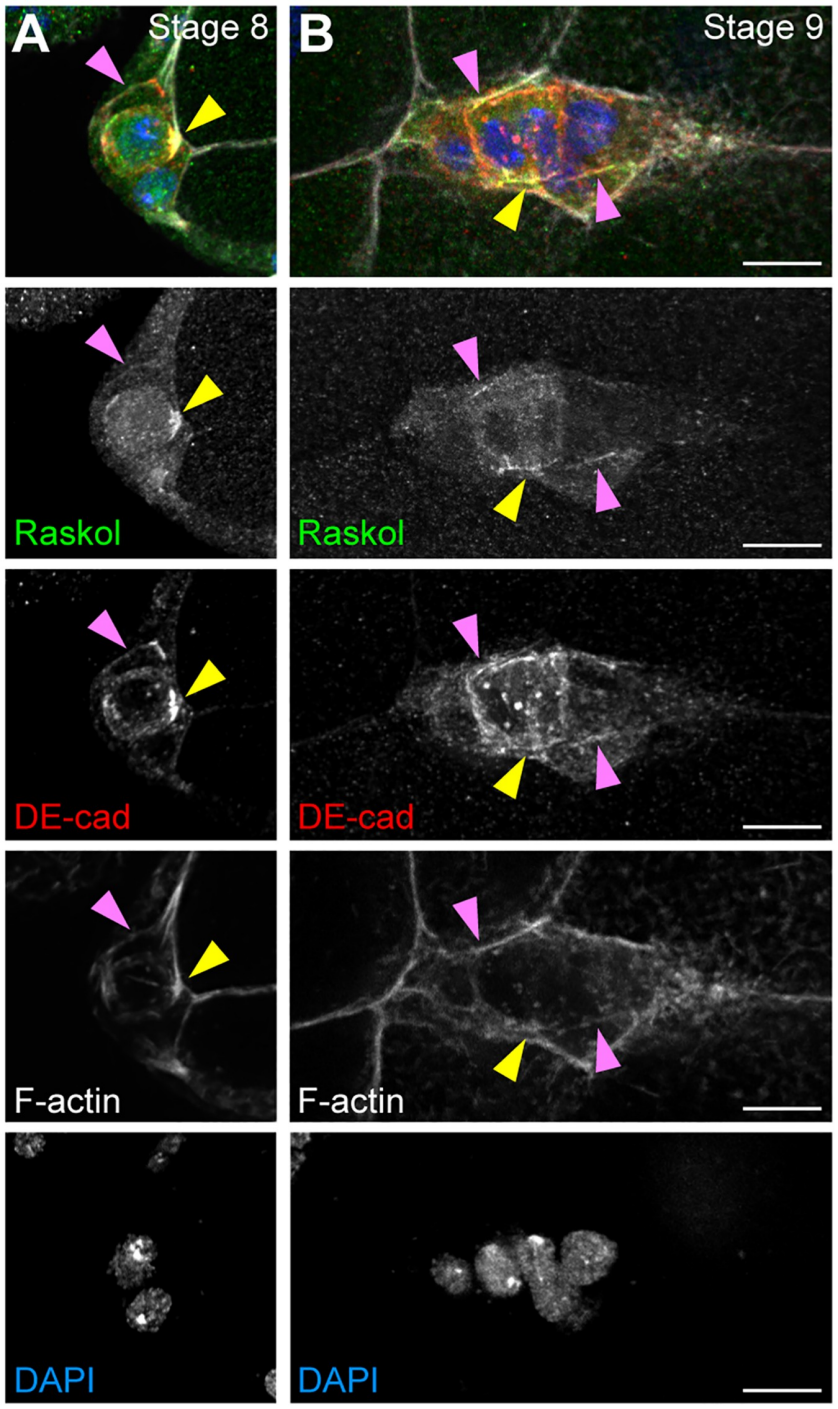
Raskol localizes to BC contacts. A-B. Stage 8 (A) and stage 9 (B) egg chambers expressing Raskol-YFP (green) and stained for DE-cad (red) and F-actin (white) and nuclei labeled with DAPI (blue). Yellow triangles indicate the apical side of PCs. Pink triangles mark cell-cell contacts. Maximum projections of deconvolved 5 μm z-stacks are shown. Scale bar in B is 10 μm and applies to A-B.

Knockdown of *CG42684* displayed the most severe cell disassociation phenotype amongst all genes tested (Fig 3). CG42684 is reported to localize at the cell cortex and is enriched specifically at the apical surface of epithelial cells in *Drosophila* embryo [55] though little is known about its molecular function in flies. The mammalian homologs of CG42684, Rasal2 and Dab2IP, also localize to the PM [56]. Interestingly, and similar to the impact we report here for CG42684, downregulation of Rasal2 disrupts E-Cadherin localization at the cell contacts [57], though the mechanism for this disruption remains poorly defined and its conservation across phyla has yet to be reported. Therefore, we wanted to determine whether CG42684, which we named “Raskol” (Russian for “to split”) associates with DE-cad along the cell membrane. Consistent with earlier localization studies, a YFP-trap stock expressing Raskol-YFP localized to the cell periphery in embryonic epidermal cells, particularly along the apical membrane (S2 Fig) [55]. We used this stock to assess Raskol colocalization with DE-cad along the BC membrane. In *Drosophila* stage 8 embryos, before BCs have delaminated, Raskol localized to the cell membrane of BCs and PCs and was enriched at the PC apical membrane (Fig 5A, S2B Fig). During migratory stages, Raskol localization persisted at the PC apical membrane and at the cell-cell contacts of BCs and PCs (Fig 5B). Immunolabeling of egg chambers with DE-cad antibody revealed colocalization between DE-cad and Raskol at the apical surface of PCs and at BC-BC contacts (Fig 5A and 5B).

Next, we determined whether Raskol colocalizes with DE-cad in other tissues that require AJ-mediated adhesion. Dorsal closure (DC) is an embryonic process in which the migrating ectoderm closes the dorsal hole [58–60]. The amnioserosa, an extra-embryonic tissue, covers the dorsal hole and contributes to ectodermal closure by providing contractile forces that pull the contralateral ectodermal sheets together [58, 61, 62]. DC requires DE-cad-mediated adhesion for ectodermal migration and fusion [63, 64]. To analyze Raskol and DE-cad dynamics, we conducted time-lapse live imaging of embryos expressing YFP-tagged Raskol and RFP-tagged DE-cad during DC. Colocalization of Raskol and DE-cad was observed both at the amnioserosa cell contacts as well as in the dorsal most ectodermal cells at the zippering interface (S3 Fig). Raskol and DE-cad colocalize in multiple *Drosophila* tissues, suggesting that Raskol may be a fundamental regulator of DE-cad.

### Raskol regulates the distribution of polarized actin protrusions

**Fig 6:**
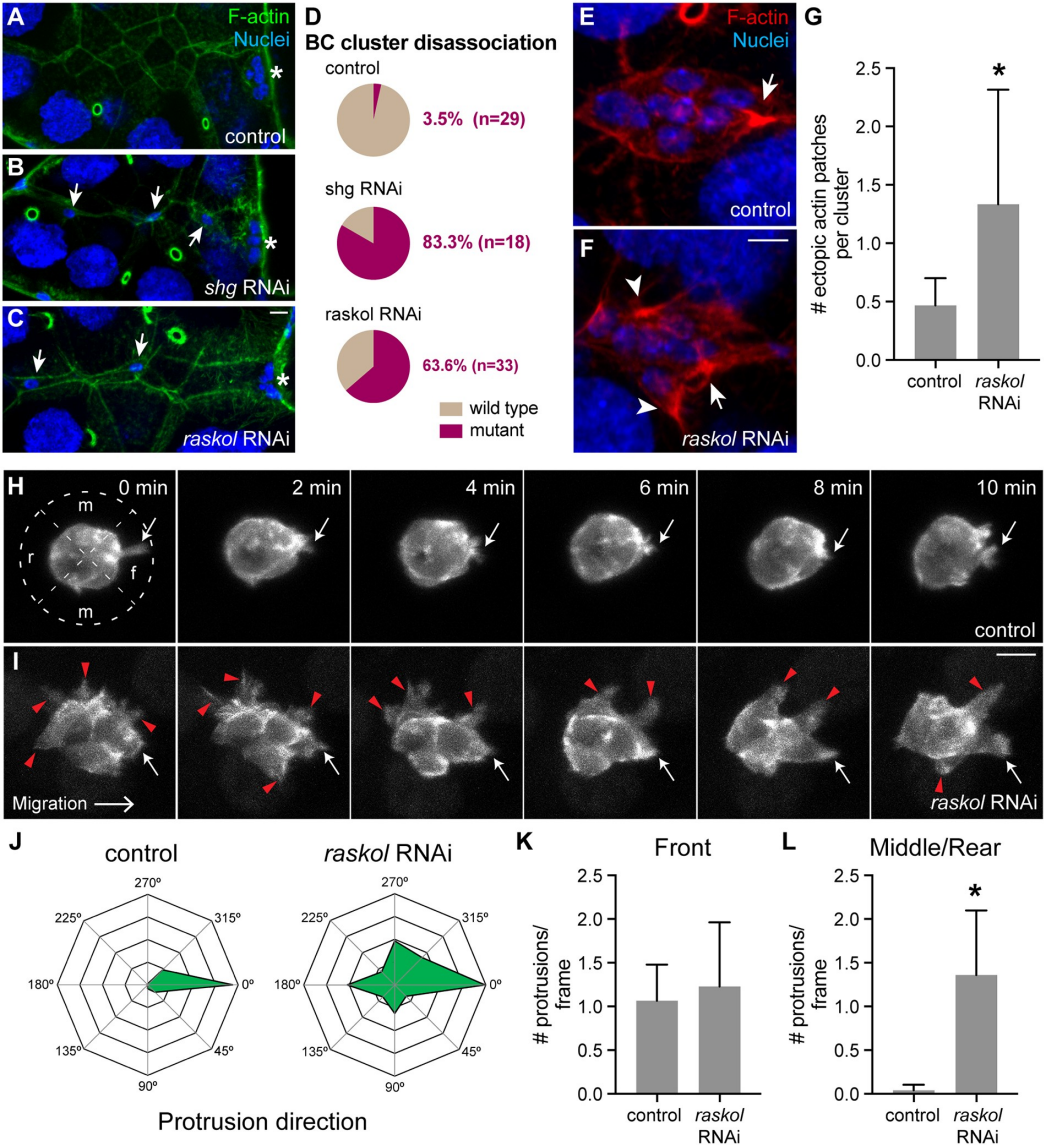
Raskol regulates actin organization in migrating BC clusters. A-C. Representative images of egg chambers stained for F-actin (green) and nuclei (blue) expressing control RNAi (A), *shg* RNAi (B) and *raskol* RNAi (C) in PCs using *upd*-GAL4. Asterisks mark the final position of the BC cluster adjacent to the oocyte. Arrows mark disassociated BC cells along the migratory path. Maximum projections of 10 μm stacks z-stacks are shown. D. Quantification of the cluster disassociation phenotype in control, *shg* and *raskol* RNAi-expressing BCs (p<0.0001). E-F. BC cluster expressing control RNAi (E) and *raskol* RNAi (F) under the control of *slbo*-GAL4 and stained for F-actin (red) and nuclei (blue). A white arrow marks the actin patch at the leading protrusion. White arrowheads mark ectopic actin patches around the BC cluster. G. Quantification of ectopic actin patches in control (n = 30) and *raskol* (n = 39) RNAi BC clusters (p<0.0001). H-I. Time-lapse images of migrating cluster expressing lifeact-GFP and control RNAi (H, n = 8) or *raskol* RNAi (I, n = 8) in BCs. White arrows mark the leading protrusion. Red triangles mark ectopic protrusions. f—front, m—middle and r—rear. J. Radar maps showing the distribution of protrusions around the BC cluster. 0° is the direction of migration. K. Number of front-oriented protrusions per frame observed in control and *raskol* RNAi expressing clusters. p = 0.71. L. Number of protrusions per frame at the middle and rear of control RNAi and *raskol* RNAi-expressing clusters (p = 0.0002). Scale bar in C is 10 μm and applies to A-C. Scale bar in panel F is 10 μm and applies to E-F. Scale bar in I is 10 μm and applies to H-I. Error bars in all graphs represent SD.

Analysis of Raskol-YFP protein localization in BC clusters revealed that cytoplasmic levels of Raskol were ~2x higher in PCs compared to BCs (S4A Fig). DE-cad levels are up-regulated in PCs relative to BCs [24], suggesting that Raskol protein expression pattern trends with DE-cad. Accordingly, *shg* and *raskol* gene expression patterns overlap during embryonic development and both peak at 6–8 hrs after egg laying (S4B Fig) [48]. To determine if Raskol is required for maintaining cell adhesion in PCs similar to BCs, we expressed *raskol* RNAi using *unpaired*-GAL4 (*upd*-GAL4) to drive expression specifically in the PCs [65]. Egg chambers were stained for F-actin (phalloidin) and nuclei (DAPI). Expression of control RNAi did not affect cluster adherence; however, *shg* RNAi expression caused cluster disassociation in ~80% egg chambers (Fig 6A, 6B and 6D), similar to a previous report [24]. Expression of *raskol* RNAi in PCs caused BC disassociation in 63% of egg chambers (Fig 6C and 6D), suggesting that Raskol and DE-cad might function together to promote BC cluster adhesion in both PCs and BCs.

Next, since Raskol contains a GAP domain and colocalizes with the cortical actin cytoskeleton in BCs, the FE, the amnioserosa and the dorsal most ectodermal cells [59, 64], we sought to determine whether it functions to regulate actin organization in BCs. We stained egg chambers expressing *raskol* RNAi in the BCs under the control of *slbo*-GAL4 driver for F-actin (phalloidin) and nuclei (DAPI) to assess F-actin distribution in the migrating cluster. In control BCs, actin accumulated at the base of protrusions typically oriented in the direction of migration (Fig 6E). In contrast, downregulation of *raskol* resulted in dramatic formation of multiple ectopic actin patches around the BC cluster (Fig 6F and 6G).

We then performed time-lapse live imaging of BC clusters expressing UAS-lifeact-GFP under the control of *slbo*-GAL4 to analyze protrusion dynamics in more detail. In control egg chambers, we observed protrusions extending primarily from the front of the migrating cluster (Fig 6H and 6J–6L; S1 Movie). In contrast, when *raskol* was downregulated, protrusions extended indiscriminately around the cluster (Fig 6I–6L; S2 Movie), consistent with our previous observations. Interestingly, the number of front-oriented protrusions in control and *raskol* RNAi expressing BCs did not differ significantly (Fig 6K). These data suggest that Raskol acts to restrict actin protrusions to the front of the BC cluster, which is critical to regulate BC migration. In addition, *raskol* knockdown caused BC delamination defects (S3 Movie) and cluster disassociation (S4 Movie) thereby confirming its importance in controlling cell adhesion and providing initial mechanistic insight into its role in regulating actin dynamics.

## Discussion

We combined evolution-guided bioinformatics with classical RNAi-based screening in *Drosophila* to identify regulators of E-cadherin-mediated cell adhesion. Our screen uncovered both established and novel regulators of DE-cad function during BC migration. We demonstrated that one hit, the previously uncharacterized GAP domain containing protein Raskol, colocalizes with DE-cad and regulates polarized actin dynamics in migrating BCs.

### ERC analysis reveals an evolutionary relationship between core components of the AJ

Core AJ components DE-cad, α-cat and p120ctn display high ERC values relative to one another, suggesting that the AJ complex has coevolved under selective pressure. α-Cat provides the mechanical link between the cadherin-catenin complex and the actin cytoskeleton. Actin linkage is believed to be the original, ancestral function of the adherens junction that provided the foundation for multicellularity [9], so it is not surprising that DE-cad and α-cat, given their functional roles, have coevolved. Interestingly, p120ctn is not essential for cadherin-mediated adhesion in flies [66], though it plays an important role in cadherin endocytosis in flies [67], similar to its established role in vertebrates [68]. Our ERC analysis suggests that shared selective pressures guided the evolution of the p120cat and DE-cad complex, and we speculate that these pressures may have shaped the range of p120cat functions in higher vertebrates.

Notably, no significant ERC relationship was observed between DE-cad and Arm or DE-cad and other secondary AJ complex proteins. While Arm is a core component of the AJ, it also functions as a key transcription factor in the Wnt signaling pathway [28, 29, 69]. We speculate that Arm function in Wnt signaling placed additional evolutionary pressures and altered its ERC signature relative to the other AJ proteins. Similarly, neither Cno nor Vinc showed a strong ERC relationship to AJ proteins, possibly reflecting their individual roles in AJ-independent processes [31, 70].

A number of proteins identified in the DE-cad ERC analysis have been implicated in regulating cell adhesion (Fig 1, Table 1). However, many ERC-identified proteins have not been functionally associated with DE-cad, the AJ or cell adhesion. A previous genomic RNAi screen conducted in *Drosophila* identified multiple regulators of DE-cad [43]. Notably, there was little overlap between the two screens. This highlights the potential of ERC analysis as an alternative, unbiased approach to generate a target protein/gene list based solely on the evolutionary rate comparison. However, it is important to conduct secondary screens to validate the function of the targets in a relevant biological system to eliminate false positives [16, 18, 19]. Also, ERC analysis cannot predict where (e.g., tissue type) or when (e.g., developmental stage) a putative interaction will occur. Refinement of the DE-cad ERC list based on spatio-temporal expression data can further eliminate false positive hits [19]. Nonetheless, a major advantage of ERC analysis is that protein/genes that would otherwise not arise in a functional or associative screen can be identified.

### DE-cad and its regulators are required for BC migration

BC migration requires coordinated regulation of adhesion and motility [23, 71, 72] and is a good system for testing genes that regulate DE-cad. BC migration is also an *in vivo* model for metastasis since many morphological characteristics of BCs resemble the invasive behaviour of metastatic cell clusters [72, 73]. However, in contrast to most models of epithelial-to-mesenchymal transition, the detachment of BC cluster requires upregulated levels of DE-cad [23, 24]. DE-cad-mediated adhesion between BCs and NCs is required for cluster polarization and directional migration, whereas adhesions between BCs and PCs are required for cluster adherence during migration [24, 25, 74]. Thus, BC migration is a useful system for genetic studies of cell adhesion and offers an opportunity to explore the role of adhesion genes in a relevant disease model [72, 73].

Our secondary genetic screen of DE-cad ERC hits revealed potential roles for a number of genes in BC migration, including kinases, GTPase regulators, transcription factors and cytoskeletal proteins. A small number of hits have putative or established roles in regulating DE-cad and/or AJs. For example, PDZ-GEF was shown to colocalize with DE-cad and function through the GTPase Rap1 to regulate DE-cad at the cell membrane [34, 35]. Hrb98DE is an RNA-binding protein that regulates DE-cad mRNA processing [32, 33]. Mammalian homologs of the small GTPase Rab5 regulate E-Cad trafficking [40, 42]. The mammalian homolog of transcription factor CG16952, Btbd7, regulates E-Cad expression [38, 39]. Additionally, RhoGAPp190, Stim, Appl and Rab5 were also identified in functional and proteomics screens of E-Cad [43, 44]. We also identified numerous proteins that have not been linked to DE-cad, including Raskol, CG11593, Babo and Zormin. Of these, we found that Raskol, CG11593 and Babo regulate DE-cad levels at BC-BC contacts (Fig 4). Using DE-cad ERC analysis and BC migration as a genetic model we have identified multiple novel proteins that function to regulate DE-cad-mediated cell adhesion in *Drosophila*.

### Raskol is a putative regulator of cell adhesion, polarity and actin dynamics

Downregulation of *raskol* caused severe BC cluster disassociation suggesting that Raskol is a critical regulator of BC adhesion. Consistent with this, Raskol colocalized with DE-cad in multiple cell types and knockdown of *raskol* reduced DE-cad levels at BC-BC cell contacts. Like *shg*, *raskol* is upregulated in PCs relative to BCs and NCs. The encoded protein contains a conserved GAP domain that displays homology towards Ras- and Rho-GAPs, a plekstrin homology (PH) domain and a C2 domain that likely promote its membrane localization [75]. This suggests that, by colocalizing with DE-cad, Raskol regulates adhesive strength between BCs to maintain cluster adhesion during detachment from the FE and subsequent migration. The mammalian homologs of Raskol, Rasal2 and Dab2IP, were identified in a screen for RasGAP tumour suppressors [75] and are frequently downregulated in multiple types of cancer cells [57, 76–79]. Rasal2 and Dab2IP are capable of inactivating Ras through inducing GTP hydrolysis through their GAP domain and their downregulation leads to Ras overactivation [80–82]. Furthermore, inactivation of Rasal2 promotes invasive behaviour in a cell migration assay suggesting that Rasal2 has a conserved role in regulating cell adhesion and protrusive behaviour in mammals [75]. Dab2IP was identified in cadherin proximity biotinylation screens in mammalian epithelial cells [44] and mouse neonatal cardiomyocytes [83], further suggesting that the Rasal2/Dab2IP/Raskol family of proteins regulate AJ biology. Nonetheless, the mechanism of their function remains unclear.

Our study offers potential insight into Raskol function during collective migration. Epidermal growth factor receptor (EGFR) and PDGF- and VEGF-related receptor (PVR) localize to the leading edge of BC clusters and respond to a presumed gradient of guidance cues originating from the oocyte [71, 84–86]. The BC with the highest levels of EGFR/PVR activation becomes the leader cell and relays a signal to neighboring BCs through the DE-cad adhesion complex to inhibit protrusion formation at the sides or rear of the cluster [24]. Interestingly, *gurken*, which encodes one of the four ligands for EGFR [85, 87, 88], also appeared on the DE-cad ERC list (ERC value 0.62, Table 1) as did its receptor *Egfr* (epidermal growth factor receptor, ERC value 0.36; S1 Table). The presence of both ligand and receptor suggests that EGF signaling has coevolved with DE-cad to regulate cell adhesion. The primary GTPase that functions to regulate the directional migration of BC downstream of EGFR and PVR is Rac1, a member of the Rho GTPase family of proteins. We propose that Raskol, as a GAP, may function to suppress Rac1 signalling in non-leader BCs. Rac1 is expressed in all BCs, but the leader cells exhibit higher activity due to increased activation of EGFR and PVR [24, 84, 89]. Our results show that Raskol, like EGFR, PVR and Rac1 [84, 85, 89], restricts protrusions to the front of migrating BC cluster thus ensuring unidirectional migration. Downregulation of DE-cad causes disruption in the polarized distribution of Rac1 in BC clusters, suggesting that DE-cad regulates signaling downstream of EGFR and PVR [24, 90]. Therefore, Rac1 suppression might be achieved through Raskol GAP activity since knockdown of Rac1 or Raskol produce similar protrusion phenotypes [89]. Raskol may buffer the DE-cad/Rac/actin mechanical feedback loop to regulate cell adhesion and promote collective cell migration. Whether Raskol directly regulates the GTPase activity of Rac1 remains to be explored.

Raskol localization is polarized with highest levels observed at the apical domain of ectodermal cells, FE cells and PCs. This suggests that Raskol might regulate actin dynamics at the apical domain of polarized cells. However, Raskol does not directly regulate formation of protrusions since reducing Raskol levels does not affect the prevalence of leading protrusions. We predict that in leading BCs, Raskol limits active Rac1 to the leading protrusion to induce localized actin cytoskeletal remodeling. Overall, these data highlights two potential roles of Raskol function: 1) as a regulator of cell adhesion, and 2) as a regulator of actin dynamics in migrating cluster, possibly downstream of receptor tyrosine kinase signaling. Future studies dissecting the role of Raskol and other proteins identified in this study are expected to offer insight into how they function with the AJ to regulate cell adhesion and cell migration.

## Methods and materials

### Evolutionary rate covariance analysis

ERC values were calculated from protein coding sequences from 22 *Drosophila* species: *D*. *ananassae*, *D*. *biarmpies*, *D*. *bipectinada*, *D*. *elegans*, *D*. *erecta*, *D*. *eugracilis*, *D*. *ficusphila*, *D*. *grimshawi*, *D*. *kikawaii*, *D*. *persimilis*, *D*. *pseudoobscura*, *D*. *melanogaster*, *D*. *miranda*, *D*. *mojavensis*, *D*. *rhopaloa*, *D*. *sechelia*, *D*. *simulans*, *D*. *suzukii*, *D*. *takahashii*, *D*. *virilis*, *D*. *willistoni* and *D*. *yakuba*. Protein coding sequences were downloaded from the Flybase website (http://www.flybase.org/) or the NCBI genome annotation website (https://www.ncbi.nlm.nih.gov/genome/annotation_euk/all/). Initially, coding sequences were evaluated for internal stop codons and the sequence was removed if found. For genes with multiple transcripts, the transcript with the longest sequence size was selected to represent the gene.

Orthology between genes across the multiple species were determined using the Orthofinder algorithm [91]. For each orthogroup, which are sets of genes that are orthologs and/or recent paralogs to each other, we omitted paralogous genes. Only orthogroups that had at least 6 species representation were analyzed further. Gene members of each orthogroup were aligned to each other using the PRANK aligner [92].

The multisequence alignment of each orthogroup was used by the PAML *aaml* program [93] to estimate the evolutionary rates on a single fixed species topology. The amino acid substitution model implemented in *aaml* was the empirical Whelan and Goldman (WAG) amino acid replacement matrix [94]. All other parameter choices are as in the ‘codeml_template.ctl’ file in the associated data repository. A single species topology was estimated using a supertree approach by combining individual orthogroup topologies that were estimated using RAxML [95]. Trees were combined using the matrix representation method implemented in phytools [96]. All resulting orthology assignments, raw sequences, orthologous gene alignments, and gene trees are available in the data repository linked to this article.

ERC was calculated using the branch lengths of each orthogroup tree. The overall species phylogenetic rates were normalized out for each orthogroup’s evolutionary rate, as described previously [18, 19]. Briefly, each orthogroup tree’s branch lengths were regressed against the average branch lengths across all orthogroup trees, and the residuals of the orthogroup tree’s branches were used as the relative evolutionary rates (RERs) for that orthogroup, such that a positive RER represents more evolutionary change than expected and a negative RER less. ERC was measured as the Kendall’s τ correlation coefficients between two orthogroups and their RERs. ERC was then calculated for all pairwise orthogroup combinations and was carried out for each pair on the tree containing exactly the set of species shared by that orthogroup gene pair. The R code to make these calculations and all results are available in the associated data repository.

### Gene ontology and expression analysis

DE-cad ERC candidates were subjected to gene ontology (GO) analysis using Flybase website (http://www.flybase.org/). Homologs of *Drosophila* genes in mammalian genomes were generated using Flybase (http://www.flybase.org/). A mammalian gene was considered a homolog if the gene was reported by 45% or more algorithms. Mammalian homologs were analyzed for canonical pathway and disease & function enrichment using Ingenuity Pathway Analysis tools (https://www.qiagenbioinformatics.com/products/ingenuity-pathway-analysis/).

RNAseq data of Drosophila gene expression profile was downloaded from flybase(ftp://ftp.flybase.net/releases/FB2018_03/precomputed_files/genes/gene_rpkm_report_fb_2018_03.tsv.gz). Reads Per Kilobase of transcript per Million mapped reads (RPKM) was used to represent gene expression level at different stages of development.

### *Drosophila melanogaster* strains

All GAL4, reporter TRAP and RNAi stocks were obtained from Bloomington Drosophila Stock Center (BDSC) (S4 Table). *DE-cad-GFP* and *DE-cad-mCherry* knock-in stocks were used as DE-cad reporters [49]. s*lbo*-GAL4, UAS-lifeact-GFP, UAS-LacZ stock was generously provided by Jiong Chen (Nanjing University) [97]. Fly stocks were raised on standard yeast-based media at 20°C, unless otherwise noted.

### BC migration screen

For BC migration analysis, RNAi-expressing female flies under the control of *slbo*-GAL4 were collected and transferred to vials containing fresh yeast paste and males. Flies were raised at 29°C for 1–2 days. UAS-GFP was used as a reporter for RNAi expression. Dissected ovaries were fixed in 4% paraformaldehyde in PBS for 20 mins and washed 5 times with PBS. Ovaries were mounted on microscope slides in 70% glycerol and 20 μm z-stacks were acquired on a Nikon A1 scanning confocal microscope.

### Immunostaining of egg chambers

1–2 day old females were incubated at 29°C for 1–2 days in vials with fresh yeast paste and males. Ovaries were dissected, fixed in 4% paraformaldehyde for 20 mins in PBS with 0.1% Triton-X (PBST), washed 5 times with PBST and blocked in normal goat serum (NGS) for 30 mins. For primary antibody staining, ovaries were incubated with rat anti-DE-cad (1:100, Developmental Studies Hybridoma Bank) and rabbit anti-GFP (1:100, Thermo Fisher Scientific) overnight at 4°C and washed 10 times the next day over 1 hour. Next, ovaries were incubated with Alexa Fluor dye labeled secondary antibodies (1:150, Thermo Fisher Scientific) and Alexa Fluor dye conjugated phalloidin (1:150, Thermo Fisher Scientific) for 2 hours. Egg chambers were then incubated in DAPI for 10 mins. Ovaries were washed 5 times in PBST and washed overnight and washed again 5 times next morning. Ovaries were stored and mounted on microscope slide in 70% glycerol and then imaged on a Nikon A1 scanning confocal microscope. In Fig 5, image z-stacks were first deconvolved (3D Deconvolution) in NIS-Elements (Nikon) and assembled into maximum projections for presentation.

### Live imaging of BC clusters

Male flies containing *slbo*-GAL4, UAS-lifeact-GFP and UAS-LacZ were crossed to UAS-*raskol*-RNAi females. 1–2 day old F1 females were incubated at 29°C for 1–2 days in vials with fresh yeast paste and *slbo*-GAL4, UAS-lifeact-GFP males. Ovaries were dissected for live imaging in imaging media (Schneiders’s medium, 15% fetal bovine serum (FBS) and 0.2mg/ml Insulin; Thermo Fisher Scientific) according to published protocols [22, 98]. 100 μl of imaging media containing egg chambers was transferred to poly-D-lysine coated Mattek dishes for imaging. 20 μm z-stacks (1 μm step size) covering the whole migrating border cell cluster were acquired every 2 minutes using on a Nikon A1 scanning confocal microscope.

### Live imaging of Raskol-YFP in embryos

Raskol-YFP [55] homozygous female flies were crossed to *DE-cad-mCherry* [49] homozygous males. Embryos were collected overnight on grape juice agar plates and transferred to microscope slides coated with double-sided tape. Embryos were manually dechorionated and immediately transferred to halocarbon oil on coverslips with the dorsal side facing down. Coverslips were then attached to imaging chambers using double sided tape and imaged on a Nikon A1 scanning confocal microscope.

### Quantification and statistics

Border cell migration defects were quantified as described previously [24]. Stage 10 egg chambers were analysed for each genotype. Border cell position along the migratory path was assigned into one of the following categories: 0–25% (no migration), 25–75% (delayed migration) and 75–100% (completed migration).

To quantify defects in border cell cluster adhesion, we determined the percentage of egg chambers where individual border cells had detached from the cluster.

To quantify DE-cad levels, linescans across BC-BC contacts were used to calculate the maximum pixel intensity at the contact in ImageJ. Peak values were then normalized to the peak intensity values of cell-cell contacts between NCs for each egg chamber. All quantification was performed on original, unadjusted images. One-way ANOVA followed by Mann-Whitney tests were performed to determine significance. At least 22 border cell clusters (3 cell contacts per cluster) were imaged for each genotype.

To measure cytoplasmic levels of Raskol, an ROI was drawn in the cytoplasm of polar and border cells and average intensity determined. The ROI intensity was normalized to the average cytoplasmic intensity of Raskol-YFP in nurse cells.

Ectopic actin patch number was quantified as described [97]. Actin present at the base of the leading edge protrusion was excluded from quantification. Welch’s t-test was used to compare samples and determine significance.

To quantify protrusion direction, we measured protrusions around the BC cluster in 45° increments at each frame of the movie (16 frames from 8 movies for each genotype). Protrusions between 315° and 45° angles were considered frontal protrusions; between 45° and 135° and 225° and 315° as middle protrusions; and between 225° and 135° as rear protrusions. Mann-Whitney test was performed to statistically compare the number of protrusions between samples.

## Supporting information

S1 Fig DE-cadherin levels at BC cell-cell contacts A-F. Representative images of BC clusters expressing DE-cad-GFP and UAS-RNAi constructs (A-E) or UAS-RFP (F) in BCs under the control of *slbo*-GAL4. DAPI (first column; blue in merge), DE-cad-GFP (second column; green in merge), F-actin (third column; red in merge) and merge (fourth column) channels shown. RFP channel showing BC-specific expression of slbo-GAL4 shown in F. Scale bar is 10 μm in A-F.
(PDF)

S2 Fig Raskol colocalizes with DE-cad in the FE. A-B. Egg chambers expressing Raskol-YFP (green) and stained for DE-cad (red) and F-actin (blue). Raskol is enriched at the FE apical surface where it colocalizes with DE-cad and F-actin (arrows). Individual channels correspond to the outlined box in the merged image. A. Dorsal view of an egg chamber. B. Cross-section image of an egg chamber. FE apical membrane faces the NCs. Raskol also colocalizes with DE-cad at PC contacts (arrowheads in merge). Scale bar is 10 μm in A and B.
(PDF)

S3 Fig Raskol colocalizes with DE-cad in the amnioserosa and ectodermal cells during DC. Time-lapse images of embryos expressing Raskol-YFP and DE-cad-RFP during DC. Raskol colocalizes with DE-cad at cell-cell contacts in the amnioserosa (arrows). Raskol colocalizes with DE-cad at the zippering interface of the dorsal-most ectodermal cells (arrowheads). Individual channels correspond to the outlined box in the merged image. Scale bar is 10 μm and applies to all panels.
(PDF)

S4 Fig Raskol localization and expression analysis. A. Mean cytoplasmic levels of Raskol in PCs and BCs relative to NCs. Cytoplasmic levels of Raskol were significantly higher in PCs compared to BCs according to Welch’s t-test (n = 58, p<0.0001). B. *shg* and *raskol* expression patterns display similar trends during embryonic development. RNA-seq based expression data (Reads Per Kilobase of transcript, per Million mapped reads, RPKM; obtained from www.flybase.org) from *Drosophila* embryos were plotted for *shg* and *raskol* during embryonic stages (2 hr increments). Expression of both *shg* and *raskol* peaks 6–8 hr after egg laying.
(PDF)

S1 Table Top 500 DE-cad ERC hits. (XLSX)

S2 Table Enrichment analysis of DE-cad ERC human orthologs: Canonical pathways. (XLSX)

S3 Table Enrichment analysis of DE-cad ERC human orthologs: Disease. (XLSX)

S4 Table RNAi stocks used in this study. (XLSX)

S5 Table Border cell migration and cluster disassociation data. (XLSX)

S1 Movie Border cell migration in control RNAi egg chambers. Lifeact-GFP and RNAi transgenes expressed under control of *slbo*-GAL4. 30 mins.
(AVI)

S2 Movie Border cell migration in *raskol* RNAi egg chambers. (AVI)

S3 Movie Border cell delamination defects in *raskol* RNAi egg chambers. (AVI)

S4 Movie Border cell cluster disassociation defects in *raskol* RNAi egg chambers. (AVI)
