# Supplementary figures and images for: Evolutionary rate covariation analysis of E-cadherin identifies Raskol as a regulator of cell adhesion and actin dynamics in Drosophila
Source: PLoS Genet. 2019 Feb 14;15(2):e1007720. doi: 10.1371/journal.pgen.1007720 (PMC6375579; doi:10.1371/journal.pgen.1007720)

S1 Fig

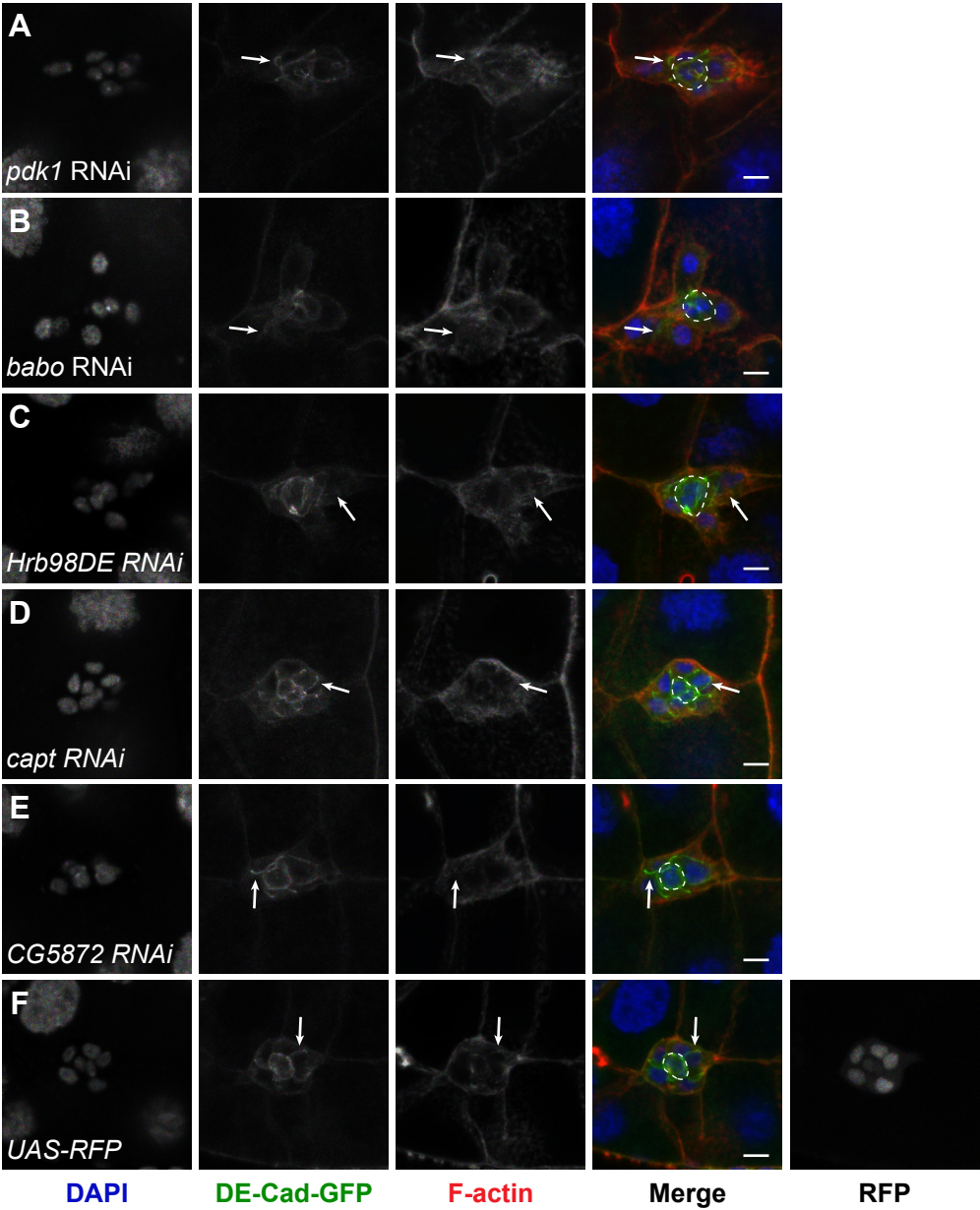

Supplement: S1 Fig — Representative images of BC clusters expressing DE-cad-GFP and UAS-RNAi constructs (A-E) or UAS-RFP (F) in BCs under the control of slbo-GAL4. DAPI (first column; blue in merge), DE-cad-GFP (second column; green in merge), F-actin (third column; red in merge) and merge (fourth column) channels shown. RFP channel showing BC-specific expression of slbo-GAL4 shown in F. Scale bar is 10 μm in A-F. (PDF) [file pgen.1007720.s001.pdf]

**S2 Fig**

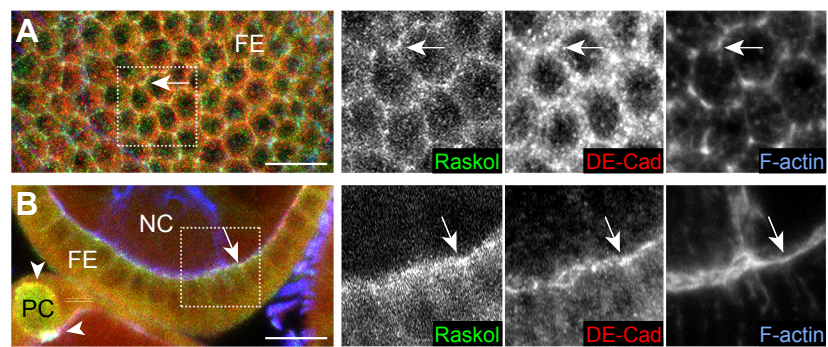

Supplement: S2 Fig — A-B. Egg chambers expressing Raskol-YFP (green) and stained for DE-cad (red) and F-actin (blue). Raskol is enriched at the FE apical surface where it colocalizes with DE-cad and F-actin (arrows). Individual channels correspond to the outlined box in the merged image. A. Dorsal view of an egg chamber. B. Cross-section image of an egg chamber. FE apical membrane faces the NCs. Raskol also colocalizes with DE-cad at PC contacts (arrowheads in merge). Scale bar is 10 μm in A and B. (PDF) [file pgen.1007720.s002.pdf]

**S3 Fig**

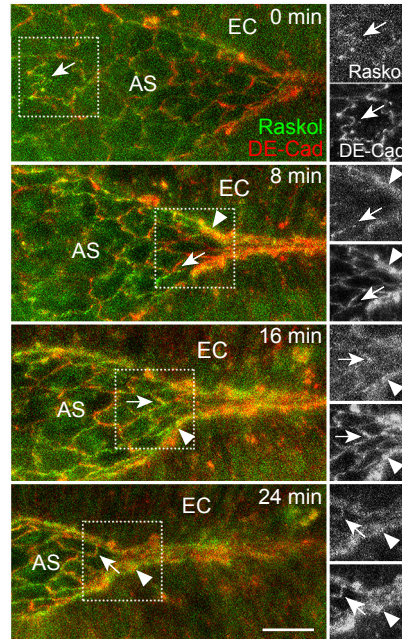

Supplement: S3 Fig — Time-lapse images of embryos expressing Raskol-YFP and DE-cad-RFP during DC. Raskol colocalizes with DE-cad at cell-cell contacts in the amnioserosa (arrows). Raskol colocalizes with DE-cad at the zippering interface of the dorsal-most ectodermal cells (arrowheads). Individual channels correspond to the outlined box in the merged image. Scale bar is 10 μm and applies to all panels. (PDF) [file pgen.1007720.s003.pdf]

S4 Fig

**A**

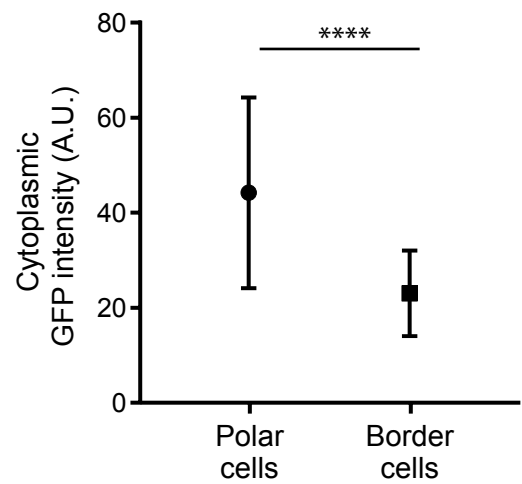

**B**

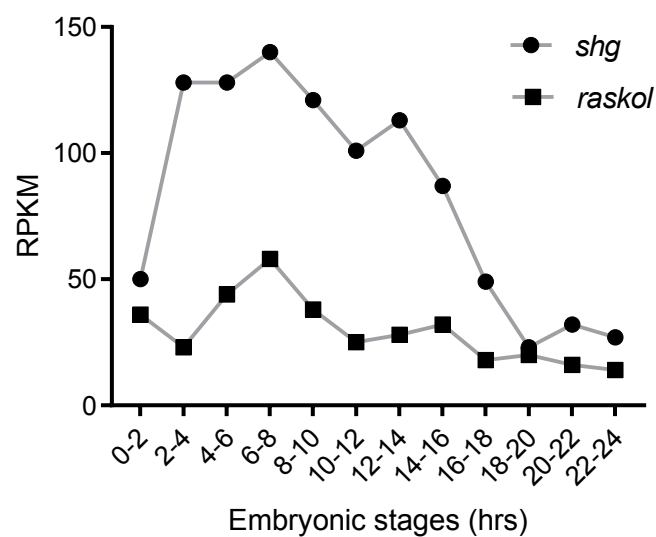

Supplement: S4 Fig — A. Mean cytoplasmic levels of Raskol in PCs and BCs relative to NCs. Cytoplasmic levels of Raskol were significantly higher in PCs compared to BCs according to Welch’s t-test (n = 58, p<0.0001). B. shg and raskol expression patterns display similar trends during embryonic development. RNA-seq based expression data (Reads Per Kilobase of transcript, per Million mapped reads, RPKM; obtained from www.flybase.org) from Drosophila embryos were plotted for shg and raskol during embryonic stages (2 hr increments). Expression of both shg and raskol peaks 6–8 hr after egg laying. (PDF) [file pgen.1007720.s004.pdf]
